# Cardioprotective Effects of the GRK2 Inhibitor Paroxetine on Isoproterenol-Induced Cardiac Remodeling by Modulating NF-κB Mediated Prohypertrophic and Profibrotic Gene Expression

**DOI:** 10.3390/ijms242417270

**Published:** 2023-12-08

**Authors:** Asma S. Alonazi, Anfal F. Bin Dayel, Danah A. Albuaijan, Alhanouf S. Bin Osfur, Fatemah M. Hakami, Shaden S. Alzayed, Ahmad R. Almotairi, Mohammad R. Khan, Hana M. Alharbi, Rehab A. Ali, Maha A. Alamin, Hanan K. Alghibiwi, Nouf M. Alrasheed, Khaled A. Alhosaini

**Affiliations:** 1 Department of Pharmacology and Toxicology, College of Pharmacy, King Saud University, Riyadh 11451, Saudi Arabia; abindayel@ksu.edu.sa (A.F.B.D.);; 2 Department of Pathology, College of Medicine, King Saud University, Riyadh 11451, Saudi Arabia

**Keywords:** GRK2, paroxetine, NF-κB, prohypertrophic genes, cardiac hypertrophy

## Abstract

Pathological cardiac remodeling is associated with cardiovascular disease and can lead to heart failure. Nuclear factor-kappa B (NF-κB) is upregulated in the hypertrophic heart. Moreover, the expression of the G-protein-coupled receptor kinase 2 (GRK2) is increased and linked to the progression of heart failure. The inhibitory effects of paroxetine on GRK2 have been established. However, its protective effect on IκBα/NFκB signaling has not been elucidated. This study investigated the cardioprotective effect of paroxetine in an animal model of cardiac hypertrophy (CH), focusing on its effect on GRK2-mediated NF-κB-regulated expression of prohypertrophic and profibrotic genes. Wistar albino rats were administered normal saline, paroxetine, or fluoxetine, followed by isoproterenol to induce CH. The cardioprotective effects of the treatments were determined by assessing cardiac injury, inflammatory biomarker levels, histopathological changes, and hypertrophic and fibrotic genes in cardiomyocytes. Paroxetine pre-treatment significantly decreased the HW/BW ratio (*p* < 0.001), and the expression of prohypertrophic and profibrotic genes Troponin-I (*p* < 0.001), BNP (*p* < 0.01), *ANP* (*p* < 0.001), hydroxyproline (*p* < 0.05), *TGF-β1* (*p* < 0.05), and *αSMA* (*p* < 0.01) as well as inflammatory markers. It also markedly decreased pIκBα, NFκB(p105) subunit expression (*p* < 0.05) and phosphorylation. The findings suggest that paroxetine prevents pathological cardiac remodeling by inhibiting the GRK2-mediated IκBα/NF-κB signaling pathway.

## 1. Introduction

Cardiovascular diseases are the leading cause of death in Saudi Arabia and worldwide, accounting for over 31% of all global deaths [1]. Cardiovascular diseases were the underlying cause of one third of deaths globally, necessitating the development of preventive strategies [2].

Pathological cardiac remodeling is a key characteristic of various cardiovascular diseases, including hypertension and ischemic heart disease [3]. The two main features of pathological cardiac remodeling are cardiac hypertrophy (CH) and cardiac fibrosis [4]. CH is an adaptive response to hemodynamic and volume stresses or high blood pressure, resulting in the stimulation of prohypertrophic signaling pathways [5,6]. Cardiac myocyte hypertrophy is associated with cardiac remodeling, which contributes markedly to the progression of cardiovascular diseases such as heart failure [6,7]. Multiple factors are involved in the development of pathological myocyte hypertrophy, such as hemodynamic stress and high levels of neurohumoral mediators, resulting in the maladaptive growth of cells without the corresponding growth of capillaries, leading to a lack of nourishment and cell injury [8]. CH is associated with elevated levels of brain natriuretic peptide (BNP) and atrial natriuretic peptide (ANP), both of which are mediated by the stimulation of pathological hypertrophy pathways, such as those mediated by angiotensin II and endothelin-1 [9,10]. Additionally, prohypertrophic genes α-smooth muscle actin (α-SMA) and β-myosin heavy chain (β-MHC) are expressed in the hypertrophic heart [10,11]. Moreover, hypertrophic cardiomyopathy is frequently associated with the progression of cardiac fibrosis, which is characterized by the accumulation of extracellular matrix (ECM), including the cardiac interstitium [12,13]. The conversion of cardiac fibroblasts to myofibroblasts is a key event that drives the fibrotic remodeling response and serves as the main source of matrix proteins. Cardiac fibrosis may involve the activation of reparative or maladaptive responses. This response is initially protective, but over time, it has negative consequences, leading to heart failure [12,13]. Transforming growth factor-β (TGF-β) plays a significant role in fibrogenesis in that it induces myofibroblast conversion and increases ECM protein synthesis [14].

Previous studies have shown that nuclear factor-kappa B (NF-κB) is elevated in the hypertrophic heart [15]. NF-κB is a transcription factor located in all nucleated cell types. It is activated in response to a diverse range of external stimuli, including inflammation, stress, and pressure [15]. The signaling pathway is mainly mediated by the p50/p65 heterodimer [15]. NF-κB translocation to the nucleus is inhibited by inhibitory-κB, the nuclear factor of kappa light polypeptide gene enhancer in B-cells inhibitor (IκB-α), which is associated with the IκB kinase (IKK) complex in the NF-κB activation pathway. NF-κB dissociates from the inhibitor IκB by phosphorylation of serine residues Ser32,36 and/or Tyr42, allowing NF-κB to translocate to the nucleus and induce the transcription of various genes, including cardiac hypertrophic genes [15].

G protein-coupled receptor kinase 2 (GRK2) is a member of the G protein-coupled receptor kinase (GRK) family and is a serine/threonine kinase responsible for the phosphorylation and regulation of G protein-coupled receptors (GPCR) [16]. GRK2 has various functions, including the regulation of β adrenergic receptors in the heart, phosphorylation of GPCR, and the activation of other enzymes and signaling pathways; it also acts as a signal transducer [16,17]. GRK2 is highly expressed in cardiomyocytes and plays a crucial role in controlling heart function through GPCR modulation and interaction with other signaling pathways [16]. The importance of GRK2 in the regulation of multiple physiological functions, particularly in the cardiovascular system, has been reported. GRK2 augmentation has been reported in cardiovascular diseases such as hypertension, myocardial infarction, and heart failure [18], suggesting that GRK2 inhibition may have cardioprotective effects [19]. Elevated GRK2 levels are associated with the incidence and progression of CH [20]. GRK2 promotes pro-inflammatory cytokine release and mediates profibrotic effects [21]. In the cytoplasm of cardiomyocytes, GRK2 phosphorylates the inhibitory IκB; phosphorylated IκB degrades rapidly and can no longer sequester NF-κB. Unbound NF-κB translocated into the nucleus and induces the expression of pro-inflammatory cytokines, such as tumor necrosis factor-a (TNFα), interleukin-1 (IL1), and IL6 [14]. Therefore, targeting GRK2 may be a promising therapeutic option for preventing the progression of cardiac myopathy. Paroxetine, a selective serotonin reuptake inhibitor (SSRI) with ATC code (N06AB), is an FDA-approved drug for the treatment of depression [22]. Paroxetine has been shown to have a selective and potent inhibitory effect on GRK2 activity by specifically binding to its catalytic domain [23,24,25]. Kowalska et al. (2021) reported that paroxetine has a promising therapeutic effect as it is able to interact with numerous molecular targets [22]. Of importance, paroxetine exhibits 50/60-fold greater selectivity for GRK2 than other GRKs, for instance, GRK1 and GRK5 [26]. Paroxetine hydrochloride was identified as well to mediate a moderate GRK2 inhibition (IC_50_ of 1.4 µM) [27]. The cardioprotective effects of paroxetine in CH, as well as its effect in mitigating associated hypertension and myocardial infarction, through the inhibition of GRK2 activity, have also been demonstrated [28]. Previous studies have shown the potential of paroxetine as a GRK2 inhibitor to reverse cardiac remodeling in experimental models of acute myocardial infarction [23,28,29]. This kinase inhibitory function influences myocyte contractility. Additionally, this inhibitory effect is limited for paroxetine and have not been shown with SSRI compound [24]. Thus, paroxetine or its derivative could be investigated for treatment of heart diseases. However, there are no published reports characterizing the cardioprotective effects of paroxetine on GRK2-mediated NF-κB signaling pathways. Thus, in this study, we aimed to investigate the molecular mechanism underlining the cardioprotective effect of paroxetine in an animal model of CH, particularly focusing on its effect on GRK2-mediated IκBα/NF-κB-regulated expression of prohypertrophic and profibrotic genes.

## 2. Results

### 2.1. Effects of Paroxetine on ISO-Induced Cardiac Injury, Hypertrophic, and Fibrotic Markers

**Figure 1:**
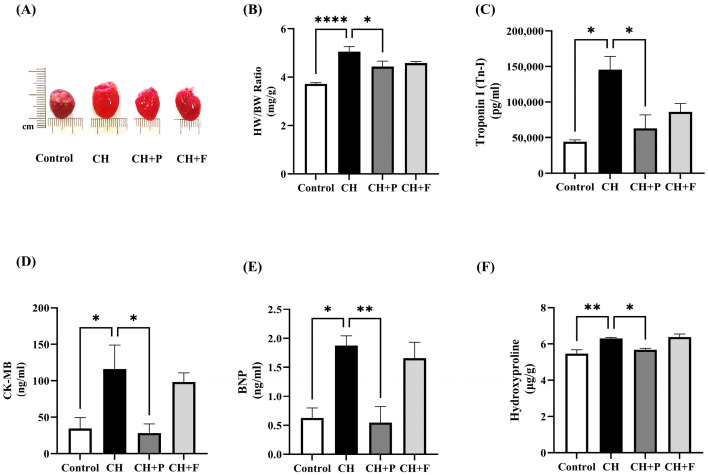
Effect of paroxetine on ISO-induced cardiac injury, hypertrophic, and fibrotic markers. (**A**) Representative pictures of hearts from control, CH, and treated rats (treated with 5 mg/kg/day paroxetine or fluoxetine). (**B**) HW/BW ratio as an indicator of cardiac hypertrophy (*n* = 6). Serum levels of cardiac injury biomarkers Tn-I (*n* = 4) (**C**) and CK-MB (*n* = 6) (**D**), cardiac hypertrophic biomarker BNP (*n* = 5) (**E**), and cardiac fibrotic marker hydroxyproline (*n* = 4) (**F**) in cardiac tissues. All data are expressed as mean ± SEM. Differences between groups were determined using one-way ANOVA followed by Dunnett’s post hoc test. Statistically significant changes compared to CH (untreated) rats are shown as * *p* < 0.05, ** *p* < 0.01, **** *p* < 0.0001. CH: cardiac hypertrophy untreated; CH + P: cardiac hypertrophy pretreated with paroxetine; CH + F: cardiac hypertrophy pretreated with fluoxetine.

**Figure 2:**
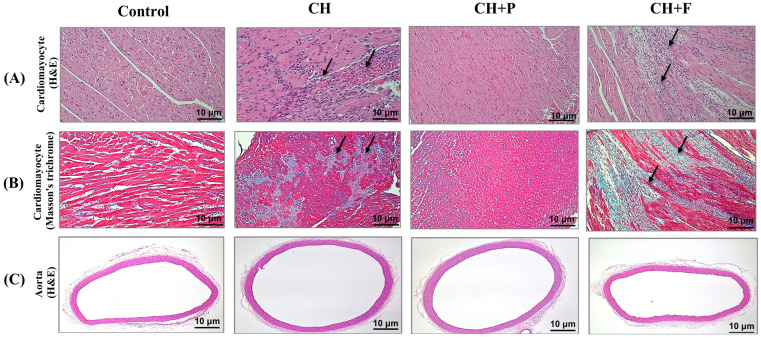
Effect of paroxetine on cardiac myocytes morphology and fibrosis development in hypertrophic heart. (**A**) H&E-stained cardiac muscle fascicle and fibers showing no inflammatory cell infiltration in the normal control group, inflammatory cell infiltration, mainly histiocytes with few lymphocytes (black arrow), in the CH (untreated) group, no inflammatory cell infiltration in the group pretreated with paroxetine (CH + P), and inflammatory cell infiltration, mainly histiocytes with few lymphocytes (black arrow) in the group pretreated with fluoxetine (CH + F) (20× power field). (**B**) Masson’s trichrome-stained cardiac muscle showing no fibrosis in the normal control group, moderate fibrosis (highlighted by blue stain) in the CH (untreated) group, mild and focal fibrosis (blue stain) in the CH + P group, and moderate fibrosis (highlighted by blue stain) in the CH + F group (20× power field). (**C**) H&E-stained rat thoracic aorta cross sections showing the three aortic layers (intima, media, and adventitia) intact with no inflammatory cell infiltration (4× power field) in the normal control. CH, CH + P, and CH + F groups. CH: cardiac hypertrophy untreated; CH + P: cardiac hypertrophy pretreated with paroxetine; CH + F: cardiac hypertrophy pretreated with fluoxetine.

To determine the effects of paroxetine on cardiac hypertrophy, the levels of cardiac injury, hypertrophy, and fibrotic markers were evaluated. As expected, the HW/BW ratio was significantly increased in hypertrophic hearts (*p* < 0.0001) (Figure 1A,B). Paroxetine pre-treatment decreased HW/BW compared to the CH untreated group (4.268 ± 0.143 vs. 5.128 ± 0.24 mg/g, *p* < 0.01). Cardiac injury biomarkers, such as Troponin-I and CK-MB, were also evaluated (Figure 1C,D). Serum Troponin-I levels were significantly increased in hypertrophic hearts (*p* < 0.001); paroxetine pre-treatment decreased Troponin-I compared to the CH untreated group (52,578 ± 14,697 vs. 145,334 ± 7638 pg/mL, *p* < 0.001). Similarly, serum CK-MB levels were significantly increased in hypertrophic hearts (*p* < 0.01); paroxetine pre-treatment decreased CK-MB compared to the CH untreated group (34.06 ± 958 vs. 106.4 ± 18.7 ng/mL, *p* < 0.01). The serum levels of the cardiac hypertrophy marker BNP were also significantly high in hypertrophic hearts (*p* < 0.01); paroxetine pre-treatment decreased serum BNP levels compared to the CH untreated group (0.546 ± 0.27 vs. 1.877 ± 0.128 ng/mL, *p* < 0.01) (Figure 1E). Hydroxyproline levels were evaluated to evaluate the effect of paroxetine on fibrotic biomarkers in cardiomyocytes. Hydroxyproline levels were significantly increased (Figure 2F) in the CH untreated group (*p* < 0.001), while paroxetine pre-treatment decreased hydroxyproline levels (5.68 ± 0.079 vs. 6.257 ± 0.064 µg/g, *p* < 0.05) (Table S1).

### 2.2. Effect of Paroxetine on Cardiac Myocyte Morphology and Fibrosis Development in Hypertrophic Heart

Figure 2A shows Hematoxylin and Eosin (H&E)-stained cardiac myocytes. It shows cardiac muscle with inflammatory cell infiltration in the CH untreated heart compared with the normal myocardium in the control. The inflammatory cells mainly include histiocytes and lymphocytes. Paroxetine pre-treatment attenuates inflammatory cell infiltration. Figure 2B shows Masson’s trichrome-stained sections of the cardiac tissue. Moderate interstitial fibrosis was observed, as highlighted by trichrome staining in the hypertrophic untreated hearts, and a similar finding was observed in fluoxetine-treated hypertrophic hearts. By contrast, hypertrophic hearts pretreated with paroxetine showed mild fibrosis with no inflammatory cell infiltration. No necrosis, granulomas, eosinophils, or giant cell infiltration were observed in any of the samples. Figure 2C shows H&E-stained aortic cross sections from the control, CH, and pretreated groups. The three aortic layers (intima, media, and adventitia) were intact with no inflammatory cell infiltration, indicating that cardiac hypertrophy did not affect aortic composition.

### 2.3. Effect of Paroxetine on NF-κB and Inflammatory Biomarkers

**Figure 3:**
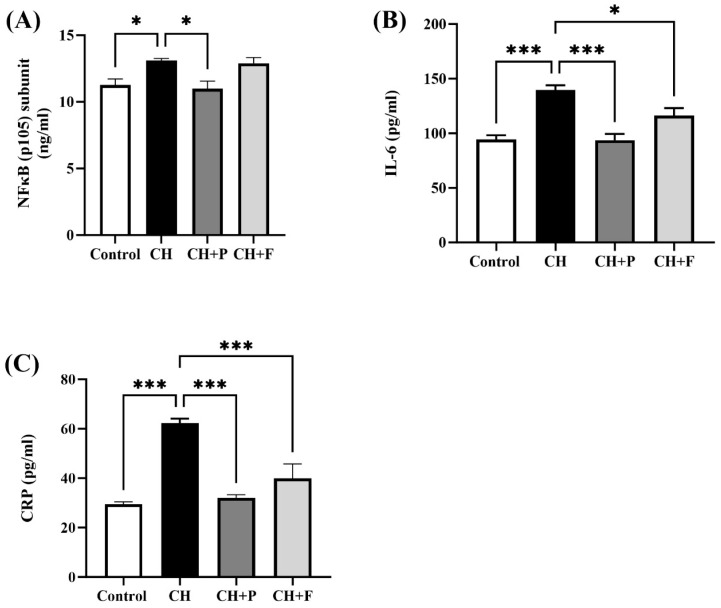
Effect of paroxetine on ISO-induced NF-κB and inflammatory biomarkers. (**A**) Level of NFκB(p105) in myocardium tissues increased with ISO treatment (CH group) and was prevented by paroxetine pre-treatment (CH + P group) (*n* = 6 per group). Serum levels of inflammatory biomarkers IL6 (*n* = 4) (**B**) and CRP (*n* = 6) (**C**) increased with ISO treatment (CH group) and were prevented by paroxetine and fluoxetine pre-treatment (CH + P and CH + F groups). All data are expressed as mean ± SEM. Differences between groups were detected using one-way ANOVA followed by Dunnett’s post hoc test. Statistically significant differences between groups are shown as * *p* < 0.05, *** *p* < 0.001. CH: cardiac hypertrophy untreated; CH + P: cardiac hypertrophy pretreated with paroxetine; CH + F: cardiac hypertrophy pretreated with fluoxetine; NFκB: Nuclear factor-kappa-light-chain-enhancer of activated B-cells; IL6: interleukin 6; CRP: C-reactive protein.

**Figure 4:**
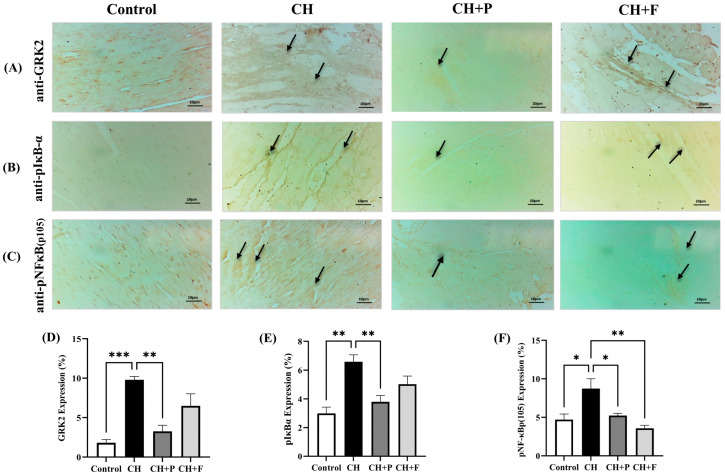
Effect of paroxetine on GRK2, pIκBα, and pNF-κBp(105) expression. (**A**) Photomicrographs showing normal GRK2 expression in the control group, increased expression in the CH (untreated) group (black arrow), reduced GRK2 expression in the CH + P group, and no marked change in the increased GRK2 expression in the CH + F group (black arrow). (**B**) Photomicrographs showing normal pIκBα expression in the control group, increased expression in the CH (untreated) group showing (black arrow), reduced expression in the CH + P group, and mild reduction of the increased pIκBα expression in the CH + F group (black arrow). (**C**) Photomicrographs showing normal pNFκBp(105) in the control group, increased expression in the CH (untreated) group (black arrow), and reduced expression in the CH + P and CH + F (black arrow). (magnification 400×). Semi-quantification results for GRK2 (**D**), pIκBα (**E**), and pNF-κBp(105) (**F**) immunoreactivity (using ImageJ software). Data are expressed as mean ± SEM (*n* = 3 different analyses per group). Statistical analyses were performed using one-way ANOVA and Tukey’s multiple comparison post hoc test; * *p* < 0.05, ** *p* < 0.01, *** *p* < 0.001 compared to the CH group. CH: cardiac hypertrophy untreated; CH + P: cardiac hypertrophy pretreated with paroxetine; CH + F: cardiac hypertrophy pretreated with fluoxetine.

NFκB(p105) subunit levels were significantly increased in the myocardium of hypertrophic rats than in control rats (13.11 ± 0.14 vs. 11.26 ± 0.45 ng/mL, *p* < 0.05) (Figure 3A); paroxetine pre-treatment reduced NFκB(p105) subunit levels compared to the CH untreated group (11 ± 0.55 vs. 13.11 ± 0.14 ng/mL, *p* < 0.05), while no change was seen in fluoxetine pre-treatment group. Serum levels of inflammatory biomarkers CRP and IL6 were also evaluated (Figure 3B,C). Serum IL6 levels were significantly increased in the CH untreated and pretreated groups than in the control group (139.7 ± 4.27 vs. 94.39 ± 3.79 pg/mL, *p* < 0.001); paroxetine pre-treatment decreased IL6 levels compared to the CH untreated group (93.55 ± 5.8 vs. 139.7 ± 4.27 pg/mL, *p* < 0.001). Similarly, pre-treatment with fluoxetine decreased IL6 levels compared to the CH untreated group (116.1 ± 6.86 vs. 139.7 ± 4.27 pg/mL, *p* < 0.05). Moreover, serum CRP levels were significantly increased in the CH untreated and pretreated groups than in the control group (62.29 ± 1.8 vs. 29.47 ± 0.957 pg/mL, *p* < 0.001), paroxetine pre-treatment decreased CRP levels compared to the CH untreated group (32.07 ± 1.24 vs. 62.29 ± 1.8 pg/mL, *p* < 0.001). Likewise, pre-treatment with fluoxetine decreased CRP levels compared to the CH untreated group (39.99 ± 5.75 vs. 62.29 ± 1.8 pg/mL, *p* < 0.001) (Figure 4C) (Table S2).

### 2.4. Effect of Paroxetine on GRK2, pIκBα, and pNF-κB(p105) Expression

**Figure 5:**
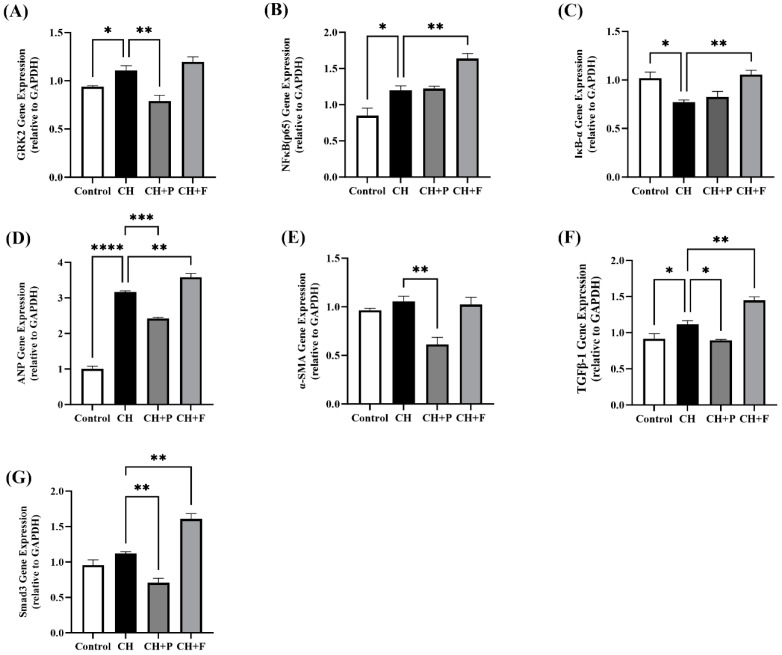
Effect of paroxetine on prohypertrophic and profibrotic gene expression. Myocardium tissue gene expression of *GRK2* (**A**), *NFκB* (p65) subunit (**B**), and *IκBα* (**C**). Expression of prohypertrophic and profibrotic genes, namely *ANP* (**D**), *α-SMA* (**E**), *TGF-β1* (**F**), and *Smad3* (**G**). All data are expressed as mean ± SEM (*n* ≥ 3). Differences between groups were detected using a one-way analysis of variance (ANOVA) followed by Dunnett’s post hoc test. Statistically significant differences between groups are indicated as * *p* < 0.05, ** *p* < 0.01, *** *p* < 0.001, and **** *p* < 0.0001. CH, cardiac hypertrophy untreated; CH + P, cardiac hypertrophy pretreated with paroxetine; CH + F, cardiac hypertrophy pretreated with fluoxetine.

Immunohistochemistry for GRK2 expression revealed increased GRK2 expression in the injured myocardium (Figure 4). Paroxetine pre-treatment reduced GRK2 expression levels (Figure 4A). Moreover, induction of cardiac injury by ISO led to a significant increase in pIκBα expression, but this increase was attenuated by paroxetine and fluoxetine pre-treatment (Figure 5B). Similarly, pNFκB(p105) expression increased in the injured myocardium by decreased paroxetine pre-treatment (Figure 4C).

### 2.5. Effect of Paroxetine on Prohypertrophic and Profibrotic Gene Expression

Cardiac prohypertrophic and profibrotic gene expression in the various groups is shown in Figure 5. *GRK2* gene expression was significantly increased in hypertrophic hearts (*p* < 0.05); compared to the CH untreated group, paroxetine pre-treatment prohypertrophic and profibrotic gene expression (*p* < 0.01); however, this effect was not observed with fluoxetine (Figure 5A). *NFκB* (p65) subunit gene expression was significantly increased in hypertrophic hearts (*p* < 0.05), non-significantly increased in paroxetine pretreated hearts, and significantly increased in fluoxetine pretreated hearts (*p* < 0.01) (Figure 5B). *IκBα* gene expression was significantly reduced in hypertrophic hearts (*p* < 0.05); however, no change was seen in paroxetine pretreated hearts, while it increased in the fluoxetine pre-treatment group compared to the CH untreated group (*p* < 0.05) (Figure 5C). Moreover, the gene expression of the cardiac hypertrophy marker *ANP* was significantly increased in the CH untreated group (*p* < 0.0001); the paroxetine pre-treatment group showed reduced *ANP* gene expression compared to the CH untreated group (*p* < 0.001) (Figure 5D). Similarly, the gene expression of the cardiac profibrotic marker *TGF-β1* was significantly increased in the CH untreated group (*p* < 0.05); the paroxetine pre-treatment group showed reduced *TGF-β1* gene expression compared to the CH untreated group (*p* < 0.05) (Figure 5F). Furthermore, paroxetine pre-treatment markedly reduced *Smad3* gene expression (*p* < 0.01) and *α-SMA* gene expression (*p* < 0.01) compared with untreated rats (Figure 5E,G) (Table S3).

## 3. Discussion

β1-adrenergic receptor activation, in response to chronic stimulation of the neuroendocrine system, has great effects on pathological remodeling of the heart and, ultimately, progression to heart failure [30]. GRK2 is an essential regulator of β1-adrenergic receptors and related signaling cascades [16]. In this study, we demonstrated the cardioprotective effect of paroxetine, a GRK2 inhibitor, in an animal model of CH, particularly focusing on its inhibitory effect on GRK2-IκBα modulation of NF-κB-mediated prohypertrophic and profibrotic gene expression.

Cardiac hypertrophy and fibrosis are common features associated with pathological cardiac remodeling [8,12]. We used a rat model of isoproterenol-induced cardiac hypertrophy as a noninvasive method for pathological CH induction [31]. Several cardiac biomarkers have been used as indicators of myocardial injury, including the HW/BW ratio, Troponin-I, CK-MB as biomarkers of hypertrophy, and hydroxyproline as a biomarker of fibrosis. ISO-treated rats showed a marked increase in HW/BW ratio and serum cardiac enzymes, including Tn-I and CK-MB, consistent with previous studies [32,33]. The observed increase in heart mass can be attributed to the sustained activation of cardiac ꞵ-adrenergic receptors following ISO treatment [34]. This result is consistent with our histopathological examination, in which ISO-treated rats showed left ventricular hypertrophy demonstrated by increased inflammatory cell infiltration. Furthermore, sustained activation of ꞵ-adrenergic receptors led to a marked increase in hydroxyproline levels, indicating severe cardiac collagen deposition. The presence of cardiac fibrosis was confirmed by histopathological examination, reinforcing increased collagen deposition in the hypertrophic hearts. These observations confirm the successful induction of pathological cardiac remodeling in the rat model, which is consistent with previous studies showing ISO-induced pathological, morphological, and structural abnormalities in rat hearts [35,36].

GRK2 plays a key role in the development of many cardiovascular diseases [16]. High levels of cardiac GRK2 are associated with the induction of cardiac hypertrophy and fibrosis [20,37]. Consistently, in this study, the ISO-induced CH model showed a significant increase in GRK2 expression. This increase in CRK2 expression may be attributed to the sustained activation of cardiac ꞵ-adrenergic receptors [28]. Inhibition of GRK2 can attenuate hypertrophic and fibrotic responses in cardiomyocytes [38,39]. Previous studies have reported that paroxetine has beneficial effects against pathological cardiac hypertrophy and fibrosis, compared to other existing SSRIs, because it acts as a GRK2 inhibitor [28,29,40]. Consistently, we found that paroxetine, but not fluoxetine, attenuated hypertrophic responses in ISO-treated rats by restoring the normal HW/BW ratio, reducing serum cardiac enzyme levels, and improving myocardial degeneration. Fluoxetine, another SSRI, served as a negative control in the current study and helped clarify whether the observed cardioprotective effects of paroxetine are related to GRK2 inhibition or are merely a result of the general properties of SSRI. Furthermore, our results showed that paroxetine alleviated the fibrotic response in ISO-treated rats by reducing collagen deposition. These results reinforce the cardioprotective effects of paroxetine. GRK2 can regulate the IκBɑ/NF-ĸB signaling pathway [41]. Previous studies have shown that GRK2 can bind and phosphorylate IκBɑ and, thus, activate the NF-ĸB signaling pathway [38,42]. This is consistent with our findings, as we observed that GRK2 overexpression in the heart was accompanied by increased phosphorylation of IκBɑ and NF-ĸB. To our knowledge, no previous study has evaluated the effect of paroxetine on IκBɑ/NF-ĸB activity in myocardial injury. Our study revealed for the first time the ability of paroxetine to inhibit IκBɑ and NF-ĸB activity in rats with myocardial injury. Paroxetine mediates this effect by inhibiting GRK2-mediated IκBα phosphorylation then eventually NF-ĸB activation.

GRK2 promotes inflammatory responses in cardiomyocytes by upregulating NF-ĸB signaling [41]. A previous study increased cytokine production in cardiac fibroblasts from neonatal rats through GRK2-mediated NF-ĸB activation [43]. Consistently, our results showed that GRK2 overexpression in the heart increased the inflammatory response, which is associated with increased NF-ĸB activation [43]. However, pre-treatment with either paroxetine or fluoxetine reduced ISO-induced inflammatory mediators. Only paroxetine, and not fluoxetine, significantly reduced NF-ĸB(p105) subunit expression. This finding suggests that paroxetine can prevent inflammation in cardiomyocytes by inhibiting the GRK2-mediated IκBα/NF-ĸB activation.

Cardiac hypertrophy is a feature of myocardial remodeling. GRK2 can induce hypertrophic responses in cardiomyocytes by modulating NF-ĸB signaling. A previous study indicated that GRK2 overexpression increases hypertrophic gene expression by increasing NF-ĸB activity [38]. Consistently, our results showed that GRK2 overexpression in pathological cardiac remodeling increased hypertrophic responses. This upregulatory effect in myocardial injury is associated with increased NF-ĸB expression [38]. In animal models of left ventricular hypertrophy, GRK2 inhibition attenuates cardiac hypertrophy by reducing NF-ĸB activity [38]. It is well-established that paroxetine attenuates cardiac hypertrophy by inhibiting GRK2 [28]. However, the mechanism by which paroxetine prevents cardiac hypertrophy remains unclear. Paroxetine pre-treatment decreased hypertrophic responses accompanied by decreased IκBα phosphorylation, as well as attenuated NF-κBp(105) subunit expression and phosphorylation in the myocardium. These results suggest that paroxetine attenuates cardiac hypertrophic response by inhibiting GRK2-mediated IκBα/NF-ĸB activation.

Cardiac fibrosis is another feature of myocardial remodeling. GRK2 is involved in the development of myocardial fibrosis [44]. Previous studies have shown that increased GRK2 expression in pathological cardiac remodeling increases the expression of genes related to cardiac fibrosis [28,45], which is consistent with our results. This increased expression was associated with increased NF-ĸB(p65) and decreased *IĸBα* expression, suggesting that GRK2 can cause myocardial fibrosis by activating the NF-ĸB signaling pathway. Paroxetine pre-treatment reduced fibrotic genes in cardiomyocytes, with a significant reduction in NF-ĸB activation. These results further reinforce that paroxetine prevents myocardial fibrosis by inhibiting GRK2-mediated IκBα/NF-ĸB activation.

One of the limitations of the current study was that the evaluation of target genes was performed at the gene level. Further investigation could be applied at the protein level as the gene expression is not absolutely correlated with the protein levels. Therefore, immunoblotting might be essential for further confirmation of the underlying mechanism of cardiac remodeling. An additional limitation of the current study is a lack of the physiological outcomes of the heart functions, which might be crucial for understanding the pathophysiology underlying cardiovascular disease. One of the reasons for not assessing the heart functions is that our study focused primarily on the target molecular mechanism rather than on the evaluation of clinical presentation. In a future study, measuring the physiological outcomes of heart functions could be applied. Additional investigation is required regarding the effect of paroxetine on various models of pathological cardiac remodeling, such as transverse aortic constriction and pressure overload-induced hypertrophy. Another limitation is that the effect of paroxetine on the GRK2-mediated IκBα/NF-ĸB signaling pathway was investigated in an animal model. Further validation is suggested by investigating its effect using primary cardiomyocytes or H9c2 cell lines for further confirmations of underlying molecular mechanisms.

## 4. Materials and Methods

### 4.1. Drugs and Chemicals

Isoproterenol (ISO) (cat#15627) was purchased from Sigma-Aldrich (St. Louis, MO, USA). Cardiac biomarkers troponin T (Tn-T) (cat# cat#SEKR-0048), creatine kinase MB (CK-MB) (cat#SEKR-0059), BNP (cat#SEKR-0058), hydroxyproline (cat#BC0255), C-reactive protein (CRP) (cat#SEKR-0017) and IL6 (cat#ab119548) enzyme-linked immunosorbent assay kit (ELISA) kits obtained from Solarbio Life Sciences (Tongzhou District, Beijing, China) and Abcam (Biotechnology Inc., Northants, UK), respectively. Primary antibodies, including anti-GRK2 (cat#sc-13143), anti-IκBα(pSer32/S36) (cat#ES1354), and anti-NFκB(p105)(Ser932) (cat#ES1367) were purchased from Santa Cruz Biotechnology (Dallas, TX, USA) and ELK Wuhan Biotechnology (Denver, CO, USA) respectively. All primers were purchased from Macrogen (Seoul, Republic of Korea). En Turbo^TM^ Syber Green supermix (cat#EQ001) was purchased from MyBioSource Life Science (San Diego, CA, USA). Other chemicals and reagents of analytical grade were supplied by standard commercial stores.

### 4.2. Animals

Twenty-four adult male albino Wistar rats (200–250 gm) were supplied by the Animal Care Center of the College of Pharmacy, King Saud University (KSU), Riyadh, Saudi Arabia. The rats were housed in optimal conditions with an air-conditioned room (25 ± 1 °C), 12 h light/dark cycle, humidity 60%, and allowed free access to feed and tap water ad libitum. The experimental design followed the guidelines of the Ethics Committee of King Saud University of Experimental Animals, which was in accordance with the international standards for the handling of experimental animals [Ethical Approval no. KSU-SE-22-61 (10 October 2022) and KSU-SE-22-96 (20 December 2022)].

### 4.3. Experimental Animal Design

**Figure 6:**
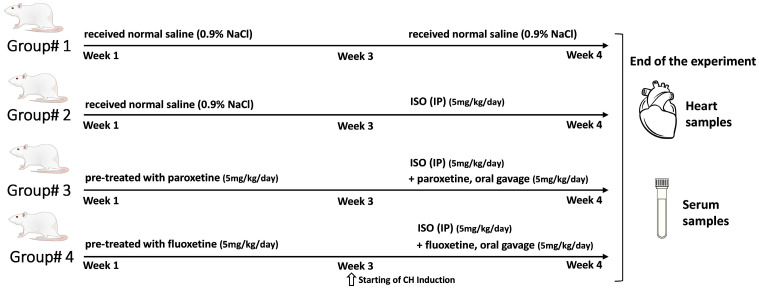
Schematic diagram representing the design and timeline of the experimental procedure. After 7 days of adaption, 24 rats were divided randomly into four groups (*n* = 6 per group): The control group, cardiac hypertrophy untreated group (CH), cardiac hypertrophy pretreated with paroxetine group (CH + P), and cardiac hypertrophy pretreated with fluoxetine group (CH + F). At the end of the experiment, serum samples were collected, and heart tissue samples were harvested.

The rats were divided into four groups (six rats per group) (Figure 6). CH was induced by daily intraperitoneal (IP) injection of ISO. ISO was dissolved in normal saline and injected (5 mg/kg) for 7 days [46]. The control rats received normal saline alone. The remaining rats were pretreated with either paroxetine or fluoxetine as follows:

Group 1: Control rats received normal saline (0.9% NaCl; IP) daily.

Group 2: CH untreated rats received normal saline (0.9% NaCl; IP) for 3 weeks, followed by CH induction with ISO (IP) for 7 days.

Group 3: CH rats pretreated with paroxetine 5 mg/kg/day by oral gavage [28] for 3 weeks, followed by CH induction with ISO (IP) for 7 days.

Group 4: CH rats pretreated with fluoxetine (as a negative control) 5 mg/kg/day by oral gavage [29] for 3 weeks, followed by CH induction with ISO (IP) for 7 days.

At the end of the experiment, all the rats were fasted overnight, anesthetized using gradually increasing concentrations of carbon monoxide gas, and euthanized. Trunk blood was collected and centrifuged at 3000 rpm for 15 min. The serum was separated and stored at −80 °C for investigation of serum cardiac injury and inflammatory markers. The heart tissue samples were rapidly isolated and weighed for determining CH. Part of the heart tissue was stored at −80 °C for molecular analysis. Another portion of heart tissue was fixed in 10% formalin for histopathological analysis.

### 4.4. Evaluating of Heart Weight/Body Weight Ratio (HW/BW)

After isolating the heart, the tissues were washed with normal saline and weighed. The HW/BW ratio was calculated by dividing the heart weight (mg) by the final body weight (g) for determining CH development.

### 4.5. Examination of Cardiac Injury, Hypertrophic, and Fibrotic Markers

Serum levels of cardiac injury biomarkers, including troponin 1 (Tn-I) and creatine kinase MB (CK-MB), were evaluated using a rat enzyme-linked immunosorbent assay kits (ELISA) (Solarbio Life Sciences, Tongzhou District, Beijing, China) according to the manufacturer’s instructions. BNP level was assessed as a cardiac hypertrophic biomarker using a rat BNP ELISA kit (Solarbio Life Sciences) according to the manufacturer’s instructions. Cardiac hydroxyproline levels were determined as an important marker reflecting the degree of collagen fibrosis in the cardiac tissue. Collagen disposition was measured using a modified version of the hydroxyproline (HYP) activity assay kit as previously described [47]. Briefly, equal weights (0.2 g) of cardiac ventricle tissue from at least four different rats in each group were homogenized and used for the detection of hydroxyproline, and a standard curve was generated according to the manufacturer’s instructions. The colorimetric results were read using a BioTek Microplate Reader at a wavelength of 560 nm. The total levels of hydroxyproline (µg/g) were determined by linear regression analysis and compared with known concentrations of standards.

### 4.6. Evaluation of NF-κB and Inflammatory Biomarkers

Myocardium tissue levels of the NFκB(p105) subunit were evaluated using a rat enzyme-linked immunosorbent assay kit (ELISA) (Solarbio Life Sciences). Briefly, equal weight (0.2 g) of cardiac ventricle tissue from at least four different rats in each group was homogenized and used for the detection of NFκB(p105) subunit levels according to the manufacturer’s instructions. Furthermore, serum levels of inflammatory biomarkers, including IL6 and C-reactive protein (CRP), were assessed using a rat ELISA kit (Solarbio Life Sciences) according to the manufacturer’s instructions. Each marker level was detected, and the standard curve was generated according to the manufacturer’s instructions. Colorimetric outcomes were read using a BioTek Microplate Reader (BioTek Instruments, Winooski, VT, USA) at specified wavelengths of λ = 405 nm, λ = 450 nm, and λ = 540 nm for NFκB(p105), CRP, and IL6, respectively. The total levels of NFκB(p105) subunit (ng/mL), IL6 (pg/mL), and CRP (pg/mL) were determined by linear regression analysis and compared with known concentrations of standards.

### 4.7. Histopathology of Heart Tissues

Histopathological evaluation of the myocardial and aortic tissue samples was performed as previously described [48]. Initially, myocardial tissue and aortic samples were removed and fixed in 10% neutral-buffered formalin (4% formaldehyde solution) for 24 h at 25 °C. They underwent routine overnight processing, including dehydration, clearing, and infiltration, and were subsequently embedded in paraffin wax. The embedded tissues were sectioned (4–5 μm) and stained with Hematoxylin and Eosin (H&E) for morphological examination of cardiac hypertrophy and aortic morphology. To investigate cardiac fibrosis, collagen deposits in the myocardial tissue sections were detected using Masson’s trichrome staining. Slides from each group were observed, and photomicrographs were taken using a light microscope (OLYMPUS, Tokyo, Japan, Light microscope, BX51) at a magnification of 400× and 200× for the myocardium and aorta, respectively. Images were evaluated by a certified surgical pathologist.

### 4.8. Immunohistochemistry of Heart Tissues

Immunohistochemistry was performed for evaluating the cardiac expression of GRK2, IκBα(pSer32/S36), and NFκB(p105)(Ser932) as previously described [49]. Paraffin-embedded heart tissue sections (5 µm) were deparaffinized and then incubated with the following primary antibodies: anti-GRK2 (1:50) (Santa Cruz Biotechnology, Santa Cruz, CA, USA), anti-IκBα(pSer32/S36) (1:100), and anti-NFκB(p105)(Ser932) (1:100) (ELK Biotechnology Co., Wuhan, China) diluted in blocking buffer [(PBS solution and 0.3% TritonX100) and 10% fetal bovine serum] at 4 °C overnight. The sections were then washed thrice with Tris-buffered saline and incubated with a secondary biotinylated IgG antibody for 30 min. After washing, sections were incubated with ABCelite for 30 min. The sections were then treated with diaminobenzidine (DAB) and counterstained with Mayer’s hematoxylin. Slides from each group were observed, and photomicrographs were captured using a light microscope (Optika Light Microscope; Ponteranica, Italy) at a magnification of 400×. GRK2, IκBα, and NFκB(p105) expressions were semi-quantified according to a previously published protocol [50] using ImageJ (v1.51) software. The semi-quantitative values of target protein expression were expressed as a percentage of the area with immunoreactivity and corrected to the total area of the field in each image.

### 4.9. Reverse Transcription Polymerase Chain Reaction (RT-PCR)

The expression of prohypertrophic and profibrotic genes was determined in ventricular myocardium tissue. RNA was extracted (from at least three cardiac tissue samples from each group) using TRIzol reagent (Invitrogen/Life Technologies, Carlsbad, CA, USA) according to the manufacturer’s instructions. RNA samples (1 μg) were reverse transcribed under standard conditions in a 20 μL reaction using the Applied Biosystems reverse transcription kit (Invitrogen/Life Technologies, Carlsbad, CA, USA). Quantitative RT-PCR amplification of the target genes was performed using EnTurbo™ SYBR Green PCR SuperMix (ELK Biotechnology Co., Wuhan, China). The PCR primers used were obtained from Humanizing Genomics Macrogen (Seoul, Republic of Korea); the sequences of forward and reverse primers for the target genes are listed in (Table 1). The reaction conditions were standardized and optimized. The *GAPDH* gene was amplified as an internal control, and gene expression was quantified for each target gene using the 2^−∆∆Ct^ method [51].

### 4.10. Statistical Analysis

All data are expressed as the mean ± standard error of the mean (SEM). Differences between groups were analyzed by one-way analysis of variance (ANOVA), followed by an appropriate post hoc test using GraphPad Prism version 9 software (GraphPad Software, San Diego, CA, USA). The differences between the groups were considered significant at *p* ˂ 0.05.

## 5. Conclusions

In conclusion, our study highlights the key role of GRK2 in the progression of cardiac remodeling. Notably, several studies have demonstrated the protective effect of paroxetine as a GRK2 inhibitor in cardiovascular diseases [23,25,28]. The transcription factor NF-κB has a key role in cardiac remodeling pathology [58]. We showed that the activation of NF-κB in the myocardium promoted the upregulation of hypertrophic and fibrotic gene expression. Therefore, we established that paroxetine prevented β1-adrenergic receptor overstimulation-induced pathological cardiac remodeling via inhibition of GRK2 activity and subsequent GRK2-mediated IκBα/NF-ĸB signaling pathway. These findings confirmed the mechanism by which paroxetine orchestrates its cardioprotective effects. Additionally, our findings implicate GRK2 as a promising therapeutic target for cardiac remodeling. Further investigating could be applied using a genetically modified animal model to further confirm the link between GRK2 kinase activity and NF-κB-mediated prohypertrophic and profibrotic gene expression.

## Figures and Tables

**Table 1:** Forward and reverse primer used for RT-PCR.

Gene	Forward	Reverse
*GRK2*	5′-TGGCCCCGGAAGTCCTA-3′	5′-CCGCAACAACTTGAAGAGCAT-3′
*NFκB (p65)*	5′-CGCAAAAGGACCTACGAGAC-3′	5′-TGGGGGAAAACTCATCAAAG-3′
*IκBα*	5′-TGAGTACCTGGACTTGCAGAACG-3′	5′-TGTAGATGCCTCTCCAAGGATGG-3′
*α-SMA*	5′-GCGTGGCTATTCCTTCGTGACTAC-3′	5′-CGTCAGGCAGTTCGTAGCTCTTC-3′
*ANP*	5′-CAGGCCATATTGGAGCAAATC-3′	5′-CTCATCTTCTACCGGCATCTT-3′
*TGF-β1*	5′-GCTGCTGACCCCCACTGAT-3′	5′-GCCACTGCCGGACAACTC-3′
*Smad3*	5′-GGCAGGATGTTTCCAGCTA-3′	5′-GCAGTCCACAGA CCATGTCA-3′
*GAPDH*	5′-GACATGCCGCCTGGAGAAAC-3′	5′-AGCCCAGGATGCCCTTTAGT-3′

Primer sequences used for real-time PCR [23,48,52,53,54,55,56,57]. GRK2, G protein-coupled receptor kinase 2; NFκB, Nuclear factor-kappa-light-chain-enhancer of activated B-cells; IκBα, nuclear factor of kappa light polypeptide gene enhancer in B-cells inhibitor, alpha; α-SMA, alpha-smooth muscle actin; ANP, Atrial natriuretic peptide; TGF-β1, Transforming growth factor beta 1; Smad3, Mothers against decapentaplegic homolog 3; GAPDH, Glyceraldehyde 3-phosphate dehydrogenase.

## Data Availability

The original contributions presented in this study are included in the article, and all data will be made available upon further inquiries directed to the corresponding author.

## Supplementary Materials

The following supporting information can be downloaded from https://www.mdpi.com/article/10.3390/ijms242417270/s1.

## Abbreviations

ANP	Atrial natriuretic peptide
α-SMA	α-smooth muscle actin
BNP	Brain natriuretic peptide
β-MHC	β-myosin heavy chain
CH	Cardiac hypertrophy
CRP	C-reactive protein
CK-MB	Creatine kinase MB
ECM	Extracellular matrix
ELISA	enzyme-linked immunosorbent assay
GPCR	G-protein-coupled receptor
GRK	G protein-coupled receptor kinase
GRK2	G protein-coupled receptor kinase 2
GAPDH	Glyceraldehyde 3-phosphate dehydrogenase
HYP	Hydroxyproline
IκBα	Nuclear factor of kappa light polypeptide gene enhancer in B-cells inhibitor, alpha
IL1	Interleukin-1
IL6	Interleukin-6
ISO	Isoproterenol
IP	intraperitoneal
NF-κB	Nuclear factor-kappa-light-chain-enhancer of activated B-cells
Smad3	Mothers against decapentaplegic homolog 3
Tn-I	Troponin 1
TGF-β	Transforming growth factor-β
TNFα	Tumor necrosis factor-α
SSRI	Selective Serotonin Reuptake Inhibitor
