# Supplementary material for: Cardioprotective Effects of the GRK2 Inhibitor Paroxetine on Isoproterenol-Induced Cardiac Remodeling by Modulating NF-κB Mediated Prohypertrophic and Profibrotic Gene Expression
Source: Int J Mol Sci. 2023 Dec 8;24(24):17270. doi: 10.3390/ijms242417270 (PMC10743803; doi:10.3390/ijms242417270)
Supplement: Supplementary file 1 [file ijms-24-17270-s001.zip › ijms-2732036-supplementary.pdf]

International Journal of
Molecular Sciences

MDPI

Table S1. Original data for the effects Paroxetine on ISO-induced cardiac injury, hypertrophic and fibrotic markers.

		Control	CH	CH+P	CH+F
HW/BW Ratio (mg/g)	Repeat no. 1	3.76	4.71	4.91	4.67
	Repeat no. 2	3.8	4.96	4.11	4.72
	Repeat no. 3	3.85	4.79	3.86	4.62
	Repeat no. 4	3.76	5.11	4.27	4.53
	Repeat no. 5	3.52	4.89	4.16	4.46
	Repeat no. 6	3.65	6.31	4.3	4.75
		Control	CH	CH+P	CH+F
Troponin-I (pg/ml)	Repeat no. 1	46817.340	145334.000	16891.097	66966.850
	Repeat no. 2	44158.000	145334.000	47480.090	120841.810
	Repeat no. 3	41498.614	126624.976	91512.615	99757.000
	Repeat no. 4	44158.000	164042.541	96295.485	75278.443
	Repeat no. 5	00000000	00000000	10709.626	154642.000
	Repeat no. 6	00000000	00000000	52578.000	81054.833
		Control	CH	CH+P	CH+F
CK-MB (ng/ml)	Repeat no. 1	66.390	66.0461	65.1856	62.087
	Repeat no. 2	4.8338	116.100	13.1689	10.565
	Repeat no. 3	39.490	187.567	16.9854	69.280
	Repeat no. 4	4.6601	155.749	18.5460	12.127
	Repeat no. 5	29.026	90.262	28.421	103.619
	Repeat no. 6	88.033	74.128	62.087	116.947
		Control	CH	CH+P	CH+F
BNP (ng/ml)	Repeat no. 1	0.477	2.197	1.267	1.020
	Repeat no. 2	0.625	2.121	0.032	2.384
	Repeat no. 3	0.431	1.533	0.182	1.468
	Repeat no. 4	0.965	1.655	1.155	1.171
	Repeat no. 5	0.630	1.877	0.092	2.231
		Control	CH	CH+P	CH+F
Hydroxyproline (µg/g)	Repeat no. 1	4.997	6.1484	5.7140	5.9500
	Repeat no. 2	4.856	6.3900	5.8662	6.0780
	Repeat no. 3	5.620	6.2015	5.4790	6.5549
	Repeat no. 4	5.336	6.0338	5.6846	6.4661
	Repeat no. 5	00000	6.4533	00000	7.0350
	Repeat no. 6	00000	6.3170	00000	6.1960

Table S2. Effect of paroxetine on NF-κB and inflammatory biomarkers.

		Control	CH	CH+P	CH+F
NFκB (p105) subunit (ng/ml)	Repeat no. 1	10.95	13.47	10.49	12.05
	Repeat no. 2	9.68	12.84	9.90	11.63
	Repeat no. 3	12.78	13.36	9.58	14.27
	Repeat no. 4	12.32	12.76	10.66	13.99
	Repeat no. 5	10.96	13.11	12.65	12.54
	Repeat no. 6	10.87	0000	12.68	12.87
		Control	CH	CH+P	CH+F
CRP (pg/ml)	Repeat no. 1	29.127	58.718	35.185	58.7180
	Repeat no. 2	31.714	63.015	36.058	57.497

	**Repeat no. 3**	**32.291**	**56.280**	**29.127**	**31.138**
	**Repeat no. 4**	**29.701**	**67.974**	**28.841**	**29.127**
	**Repeat no. 5**	**27.982**	**65.178**	**30.850**	**32.868**
		Control	CH	CH+P	CH+F
**IL6 (pg/ml)**	**Repeat no. 1**	**96.935**	**143.209**	**106.039**	**101.148**
	**Repeat no. 2**	**86.572**	**149.080**	**100.744**	**125.801**
	**Repeat no. 3**	**90.244**	**137.219**	**85.040**	**107.894**
	**Repeat no. 4**	**103.787**	**129.115**	**82.380**	**129.560**

Table S3. Effect of paroxetine on pro-hypertrophic and pro-fibrotic gene expression.

		**Control**	**CH**	**CH+P**	**CH+F**
**GRK2 Gene Expression**	**Repeat no. 1**	**0.973**	**1.152**	**0.893**	**1.094**
	**Repeat no. 2**	**0.927**	**1.014**	**0.692**	**1.229**
	**Repeat no. 3**	**0.938**	**1.161**	**0.791**	**1.264**
	**Repeat no. 4**	**0.916**	**0000**	**0000**	**0000**
		Control	CH	CH+P	CH+F
**NFκB(p65) Gene Expression**	**Repeat no. 1**	**1.049**	**1.118**	**1.219**	**1.556**
	**Repeat no. 2**	**0.795**	**1.166**	**1.263**	**1.584**
	**Repeat no. 3**	**0.710**	**1.316**	**1.123**	**1.775**
	**Repeat no. 4**	**0000**	**0000**	**1.279**	**00000**
		Control	CH	CH+P	CH+F
**IκB-α Gene Expression**	**Repeat no. 1**	**0.859**	**0.779**	**0.911**	**1.151**
	**Repeat no. 2**	**1.150**	**0.731**	**0.723**	**1.057**
	**Repeat no. 3**	**1.012**	**0.806**	**0.845**	**0.933**
	**Repeat no. 4**	**1.058**	**00000**	**00000**	**1.079**
		Control	CH	CH+P	CH+F
**TGFβ-1 Gene Expression**	**Repeat no. 1**	**0.796**	**1.096**	**0.883**	**1.541**
	**Repeat no. 2**	**0.905**	**1.044**	**0.927**	**1.369**
	**Repeat no. 3**	**1.045**	**1.213**	**0.878**	**1.433**
	**Repeat no. 4**	**0000**	**0000**	**0.897**	**0000**
		Control	CH	CH+P	CH+F
**ANP Gene Expression**	**Repeat no. 1**	**0.865**	**3.106**	**2.470**	**3.659**
	**Repeat no. 2**	**1.036**	**3.229**	**2.434**	**3.717**
	**Repeat no. 3**	**1.116**	**3.147**	**2.366**	**3.367**
	**Repeat no. 4**	**0000**	**0000**	**0000**	**0000**
		Control	CH	CH+P	CH+F
**α-SMA Gene Expression**	**Repeat no. 1**	**0.938**	**1.062**	**0.640**	**0.884**
	**Repeat no. 2**	**0.953**	**1.145**	**0.800**	**1.056**
	**Repeat no. 3**	**1.003**	**0.965**	**0.551**	**1.133**
	**Repeat no. 4**	**0000**	**0000**	**0.464**	**0000**
		Control	CH	CH+P	CH+F
**Smad3 Gene Expression**	**Repeat no. 1**	**1.031**	**1.161**	**0.815**	**1.471**
	**Repeat no. 2**	**0.882**	**1.080**	**0.601**	**1.741**
	**Repeat no. 3**	**0.956**	**1.120**	**0.708**	**1.606**
	**Repeat no. 4**	**0000**	**0000**	**0000**	**0000**
